# Supplementary material for: Markets, Herding and Response to External Information
Source: PLoS One. 2015 Jul 23;10(7):e0133287. doi: 10.1371/journal.pone.0133287 (PMC4512694; doi:10.1371/journal.pone.0133287)
Supplement: S1 Appendix — Derivation of the effective potential both for the original Kirman dynamics and the model with external information, presented in the Eqs (8) and (20). (PDF) [file pone.0133287.s001.pdf]

Markets, herding and response to external information

Adrián Carro[*], Raúl Toral, Maxi San Miguel,

Instituto de Física Interdisciplinar y Sistemas Complejos (IFISC), CSIC-UIB, Palma de Mallorca, Spain

* E-mail: adrian.carro@ifisc.uib-csic.es

S1 Appendix: Effective potential derivation

We derive, in this appendix, the effective potential both for the original Kirman dynamics and the model with external information, presented in Eq. (8) and Eq. (20) in the main text. Let us start by restating here the definition of effective potential $U_{\text{eff}}(x)$ given in Eq. (7),

$$P_{\text{st}}(x) \equiv \mathcal{C}^{-1} \exp\left(-\frac{U_{\text{eff}}(x)}{D}\right), \tag{S1.1}$$

where $P_{\text{st}}(x)$ is the stationary state probability distribution, D is an effective noise intensity that we take as $D = h$, and the constant $\mathcal{C}^{-1}$ plays the role of a normalization factor. Note that, defined as such, the minima of this effective potential function will be attractive points of the dynamics, corresponding to maxima of the stationary state probability distribution.

For the general Fokker-Planck equation

$$\frac{\partial P_{\text{st}}(x,t)}{\partial t} = -\frac{\partial}{\partial x}\left[q(x)P(x,t)\right] + \frac{\partial^2}{\partial x^2}\left[Dg(x)^2 P(x,t)\right], \tag{S1.2}$$

the stationary distribution is found by assuming $\partial P_{\text{st}}/\partial t = 0$ and solving the resulting equation. By this means, a general effective potential [1] can be written as

$$U_{\text{eff}}(x) = -\int \frac{q(x)}{g(x)^2}dx + D\int \frac{\partial g(x)}{\partial x}\frac{1}{g(x)}dx, \tag{S1.3}$$

and, applying this definition to the Fokker-Planck equation (2) in the main text, the

particular effective potential for the Kirman dynamics is found to be

$$U_{\text{eff}}(x) = (h - a)\, \ln(1 - x^2). \tag{S1.4}$$

Note that this effective potential $U_{\text{eff}}(x)$ is not to be confused with the deterministic potential, which is always monostable and can be found by simply integrating with respect to x the deterministic part of Eq. (3).

Even though in the case with an external time varying forcing it is not possible to write a stationary state probability distribution, we assume that, at any point in time, the decay of the system to a quasi-stationary state is faster than the variation of the input signal, i.e., we assume conditions of slow driving. Therefore, we keep the previous definition of the effective potential as an approximation to this time-dependent case,

$$P(x,t) \approx \mathcal{C}^{-1} \exp\left(-\frac{U_{\text{eff}}(x,t)}{D}\right). \tag{S1.5}$$

Thereby, applying equation (S1.3) to the Fokker-Planck equation (18) leads to the particular functional form

$$U_{\text{eff}}(x,t) = (h_0 - a)\, \ln(1 - x^2) - xFi(t) \tag{S1.6}$$

for the model with arrival of external information.

References

1. San Miguel M, Toral R (2000) Stochastic effects in physical systems. In: Tirapegui E, Martínez J, Tiemann R, editors, Instabilities and Nonequilibrium Structures VI, Springer Netherlands, volume 5 of *Nonlinear Phenomena and Complex Systems*. pp. 35-127.
